# Fast-Track-Endoprothetik

**DOI:** 10.1007/s00132-023-04465-4

**Published:** 2024-01-16

**Authors:** Felix Greimel, Julia Schiegl, Matthias Meyer, Joachim Grifka, Günther Maderbacher

**Affiliations:** Orthopädische Klinik für die Universität Regensburg, Asklepios Klinikum Bad Abbach, Kaiser-Karl-V.-Allee 3, 93077 Bad Abbach, Deutschland

**Keywords:** Lokale Infiltrationsanalgesie, Frühmobilisation, Beschleunigte postoperative Erholung, Hüftgelenkendoprothetik, Kniegelenkendoprothetik, Local infiltration analgesia, Early mobilization, Enhanced recovery after surgery, Total hip replacement, Total knee replacement

## Abstract

**Einführung:**

Wesentliche Bestandteile von Fast-Track- oder Enhanced-Recovery-Programmen in der Endoprothetik sind die Optimierung der interdisziplinär-organisatorischen Abläufe sowie der prä-, intra- und postoperativen Vorgehensweisen im klinischen Alltag. Die frühpostoperative Mobilisation der Patienten ist nach Gelenkersatzoperation von zentraler Bedeutung, mit dem Ziel, Schmerzen und Komplikationen nachweislich zu vermeiden. Dieser Beitrag gibt einen detaillierten Überblick hinsichtlich der zwischenzeitlich zunehmend verbreiteten Fast-Track-Vorgehensweisen. Zudem zeigt er die klinischen Vorteile von Fast-Track für die Hüftendoprothetik im Rahmen eines randomisierten Studiendesigns evidenzbasiert auf.

**Material und Methoden:**

Es wurden 194 Patienten mit primärer HTEP nach einfach verblindeter Randomisierung in zwei Gruppen untersucht: Fast-Track (*n* = 98) oder konventioneller Behandlungspfad (*n* = 96). Als primärer Outcomeparameter war die Mobilisierung definiert, gemessen mit dem Timed-Up-and-Go-Test (TUG) in Sekunden. Sekundäre Parameter waren erreichbare Gehstrecke in Metern sowie Schmerzen auf der Numerische Rating-Skala (NRS). Alle Parameter wurden präoperativ und täglich bis zum 6. postoperativen Tag erfasst.

**Ergebnisse:**

Innerhalb der ersten postoperativen Woche wurden keine Komplikationen oder Revisionen verzeichnet. Die Fast-Track-Gruppe zeigte im Vergleich zur konventionellen Gruppe bis zum 6. postoperativen Tag signifikant bessere TUG-Werte sowie Gehstreckenergebnisse (jeweils *p* < 0,05). Bezüglich der Bewertung von Schmerzen (NRS) zeigte sich kein signifikanter Unterschied (*p* > 0,05).

**Fazit:**

Die Anwendung von Fast-Track-Konzepten in der Hüftendoprothetik kann evidenzbasiert die klinischen Ergebnisse verbessern. Diese prospektive, einfach verblindete randomisierte kontrollierte Studie konnte ein sehr gutes kurzfristiges Ergebnis mit vergleichbaren Schmerzen nach Fast-Track-HTEP im Vergleich zu einem konventionellen Behandlungspfad aufzeigen. Fast-Track-Konzepte sind hochwirksam in Bezug auf Frühmobilisierung und klinisches Ergebnis – ohne in der Frühphase ein höheres Komplikationsrisiko einzugehen.

**Graphic abstract:**

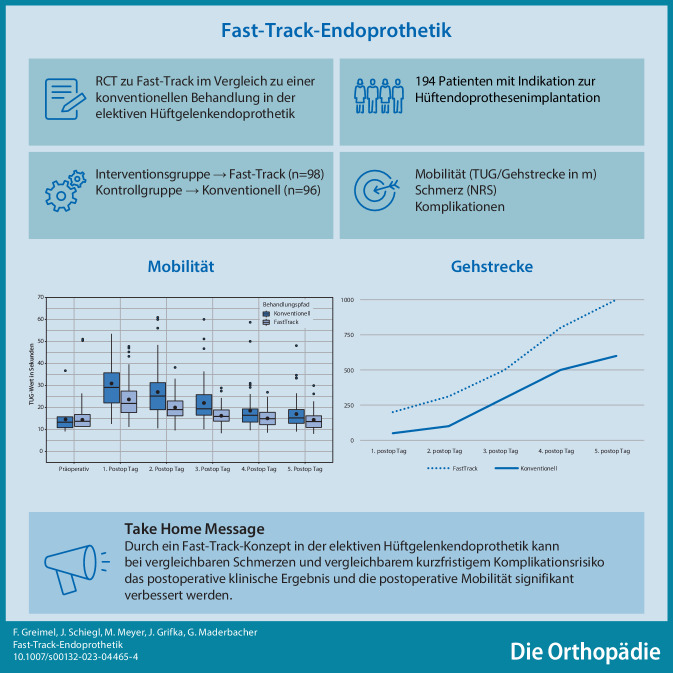

## Abstract (en)

**Introduction:**

Essential components of fast-track or enhanced recovery programs in arthroplasty are the optimization of interdisciplinary organizational processes, as well as pre-, intra- and postoperative procedures in everyday clinical practice. The early postoperative mobilization of patients after joint replacement surgery is of central importance, with the aim of avoiding pain and complications. This article provides a detailed overview of fast-track procedures that have already been established in many units. Furthermore, it demonstrates the clinical advantages of FastTrack for total hip arthroplasty (THA) in an evidence-based manner within the framework of a randomized study design.

**Material and methods:**

194 primary THA patients were examined after randomization into two single-blinded groups; Fast-Track (*n* = 98) or conventional treatment path (*n* = 96). Mobilization was defined as the primary outcome parameter, measured in seconds using the Timed Up and Go Test (TUG). Secondary parameters were the achievable walking distance in meters and pain using the numerical rating scale (NRS). All parameters were recorded preoperatively and daily until the sixth postoperative day.

**Results:**

No complications or revisions were recorded within the first postoperative week. The fast-track group showed significantly better TUG values and walking distance results compared to the conventional group until the sixth postoperative day (*p* < 0.05, respectively). There was no significant difference regarding the pain assessment (NRS) (*p* > 0.05).

**Conclusion:**

The use of Fast-Track in hip arthroplasty can evidently improve short-term postoperative clinical outcomes. This first prospective, single-blinded, randomized controlled study showed very good clinical results with comparable pain after FastTrack THA compared to a conventional treatment path. Fast-Track concepts are highly effective in terms of early mobilization and clinical outcome—without incurring a higher risk of complications in the short term.

Fast-Track-Behandlungskonzepte in der Hüft- und Knieendoprothetik haben in den vergangenen Jahren zunehmend an Bedeutung gewonnen [5]. Evidenzbasierte Vorgehensweisen im interdisziplinären Setting ermöglichen so eine wesentliche Verbesserung der frühpostoperativen Mobilisation der Patienten – auf niedrigem Schmerzniveau und unter Vermeidung von Komplikationen in der frühpostoperativen Phase.

## Grundlagen

Eine differenzierte Weiterentwicklung perioperativer Vorgehensweisen konnte in interdisziplinärer Zusammenarbeit das Gesamttrauma in der Hüft- und Kniegelenkendoprothetik reduzieren und ermöglicht eine schmerzarme Frühmobilisation und rasche Erholung der Patienten. Die rasche Mobilisation und Rekonvaleszenz der Patienten wird beim Fast-Track in der Endoprothetik in den Mittelpunkt gestellt. Hierbei handelt es sich um eine Vielzahl von organisatorischen und perioperativen Maßnahmen in interdisziplinärer Zusammenarbeit, die nur als Gesamtpaket zum Erfolg führen. Die beiden Dänen Husted und Kehlet konnten mehrere Arbeiten zum Thema Fast-Track in der Endoprothetik veröffentlichen und die Abläufe sowie Vorteile dieser Konzepte evidenzbasiert darstellen [19, 22–24]. Im Wesentlichen konnte beschrieben werden, dass sich dadurch die Morbidität und Mortalität reduzieren und eine schnelle Erholung der Patienten, eine Steigerung der Patientenzufriedenheit sowie eine Reduktion der Dauer des stationären Aufenthaltes erreichen lassen [18, 27, 28]. Im Folgenden soll stichpunktartig auf die wesentlichen inhaltlichen Bestandteile von Fast-Track in der Endoprothetik eingegangen werden.

Bei Fast-Track-Konzepten sollte auf die präoperative Verabreichung von Sedativa verzichtet werden [42]. Durch die präoperative Verabreichung von nichtsteroidalen Antirheumatika konnte eine Reduktion sowohl des Opioidbedarfs wie auch von Schmerz und Schwellung gezeigt werden [16, 19, 22, 48]. Ergänzt wird die präoperative Vorbereitung durch bereits im Vorfeld stattfindende Patientenseminare mit physiotherapeutischer Gangschule sowie eine möglichst kurze Nahrungs- und Flüssigkeitskarenz vor dem Eingriff [42].

Beim Hüft- und Kniegelenksersatz werden zunehmend Regionalnarkoseverfahren angewendet [33]. Bei Verwendung kurzwirksamer Narkoseformen bzw. Applikation von Lokalanästhetika wird über eine rasche Wiederherstellung der sensomotorischen Funktionen der Patienten die frühpostoperative Mobilisation erst ermöglicht. Postoperative Schmerzen, die Notwendigkeit von (opioidhaltigen) Schmerzmitteln sowie das Auftreten eines PONV („postoperative nausea and vomiting“) sind geringer [13, 22, 23]. Die prolongierte Verwendung von regionalen Schmerzkathetern, welche in der Kniegelenkendoprothetik weit verbreitet waren [13], kann aufgrund einer reduzierten sensomotorischen Funktion zu einer erheblichen Einschränkung der Mobilisierbarkeit mit erhöhtem Sturzrisiko führen [22] und steht daher im Widerspruch zur der in Fast-Track-Konzepten so wichtigen frühzeitigen Mobilisation. Zur Reduktion der generalisierten postoperativen inflammatorischen Antwort wird die intravenöse Applikation von Glukokortikoiden empfohlen. Zudem wurden durch die koanalgetisch-antiemetische Wirkung Vorteile hinsichtlich des Auftretens eines PONV, der Frühmobilisierbarkeit des Patienten sowie postoperativer Schmerzwerte berichtet [21, 23, 24, 49], während relevante Nebenwirkungen durch die einmalige Gabe nicht zu erwarten sind [21, 23, 24, 49]. Die Verwendung von Blasenkathetern ist aus „Komfortgründen“ weit verbreitet, jedoch mit verfahrensspezifischen Komplikationen sowie Harnwegsinfektionen und Blasenentleerungsstörungen assoziiert [2, 15]. Sie sollten daher neben einem subtilen Flüssigkeitsmanagement bei Fast-Track nicht mehr verwendet werden.

Die Verwendung von Tranexamsäure ist zwischenzeitlich in der Endoprothetik zunehmend etabliert und fester Bestandteil von Fast-Track-Konzepten. Sowohl die intravenöse, als auch die topische Applikation sind wissenschaftlich evident, wobei die topische Anwendung in Deutschland eine Aufklärung als Off-Label-Use darstellt [7, 8]. Auch die orale Applikation wird in der Literatur als effektiv beschrieben [6]. In multiplen Studien wurden positive Effekte durch die Applikation von Tranexamsäure auf den Blutverlust sowie die Transfusionswahrscheinlichkeit beschrieben [19, 22].

Auf die Verwendung von Blutsperren wird in der Fast-Track-Knieendoprothetik üblicherweise verzichtet, da Patienten durch die Blutsperre signifikant mehr Schmerzen erfahren [29], mehr Schwellung entwickeln [29] sowie von mehr Komplikationen und einer höheren Infektionswahrscheinlichkeit [1, 40] berichtet wurden. Die zunächst angenommene geringere Zementpenetration in den spongiösen Knochen durch den Verzicht auf eine Blutsperre wurde nicht bestätigt [17, 20, 26, 31].

Weiterhin ergeben sich in der Literatur Hinweise, dass gewebeschonende minimal-invasive operative Zugänge mit geringeren postoperativen Schmerzen vergesellschaftet sind sowie eine raschere Rekonvaleszenz bedingen können [9, 10, 30, 32]. Multimodale Schmerzkonzepte sollten etabliert werden. Es werden lokal und oral wirksame Analgetika eingesetzt – unter Berücksichtigung der Nebenwirkungsprofile eingesetzter Pharmazeutika, insbesondere auch der Opioide, die zurückhaltend eingesetzt werden sollten. Der Einsatz einer lokalen Infiltrationsanalgesie (LIA) in Kombination mit oralen Analgetika wird in der Knieendoprothetik favorisiert und ist auch an der Hüfte weit verbreitet [3, 14, 16, 34, 38, 41, 47]. Es wurden eine Reduktion des postoperativen Schmerzes, eine Verbesserung der Funktion sowie eine Reduktion der Dauer des Krankenhausaufenthaltes beschrieben [4, 14, 36, 44]. Im Rahmen von Fast-Track-Konzepten wird in aller Regel gänzlich auf die Einlage von Wunddrainagen verzichtet, da diese die Beweglichkeit und selbstständige Mobilisation der Patienten beeinträchtigen sowie Schmerzen verursachen können, ohne einen Effekt auf das Auftreten postoperativer Wundinfektionen, die Häufigkeit von Wundverbandwechseln, Schwellung, postoperative Schmerzen oder die Rate thrombembolischer Ereignisse zu haben [50]. Zudem lässt sich durch den Verzicht eine Senkung der Transfusionsrate erzielen [37, 38]. Ergänzend können moderne transparente und wasserdichte Pflasterverbände verwendet werden, die die sonst häufiger notwendigen Verbandswechsel überflüssig machen und das Risiko einer Wundkontamination mindern.

Zentraler Bestandteil für den Erfolg von Fast-Track-Behandlungspfaden ist die schnellstmögliche postoperative Mobilisation der Patienten unter voller Belastung und vollumfänglicher Beweglichkeit ohne Verbote, sobald die notwendigen Voraussetzungen vorliegen: Wiedererlangung der sensomotorische Funktion und kardiopulmonale Stabilität [45]. Sie stärkt hierdurch auch die Autonomie der Patienten, zudem kann die Zeit bis zum Erreichen definierter Entlasskriterien verkürzt werden [35]. Auch betagte und mobilitätsreduzierte Patienten können von der frühen Mobilisation profitieren, jedoch unter anderer Zielsetzung: Kreislaufaktivierung und Vermeidung etwaiger Komplikationen, wie Thrombose/Embolie [43]. Ergänzt wird sie mit der frühzeitigen Aufnahme von oraler Flüssigkeit und Nahrung, um die notwendige Energie rasch bereitzustellen.

Bei der Etablierung von Fast-Track ist die intensive physio- und ergotherapeutische Beübung durch entsprechend geschultes Personal relevant, der Fokus liegt vor allem auf aktiven und aktivierenden Bewegungsübungen sowie Training von Alltagsaktivitäten (ADL). Um den Übungsumfang zu erweitern, haben sich sogenannte Fast-Track-Parcours und/oder Fast-Track-Stationen als besonders hilfreich erwiesen, wobei nach entsprechender Schulung diese Parcours von Patienten in Eigenregie verwendet werden können [12]. Auch die Gruppentherapie kann eine hervorragende Motivationsstütze sein. Auf Belastungs- und Beweglichkeitslimitierungen sollte verzichtet werden, da deren Nutzen wissenschaftlich nicht belegt ist und der Patientenzufriedenheit entgegensteht [46]. Scores wie der Timed-Up-and-Go-Test (TUG) sowie definierte Entlasskriterien haben sich bewährt, den individuellen Fortschritt eines Patienten bis zur Entlassfähigkeit zu objektivieren [19, 23, 39]. Die Anwendung von Fast-Track-Konzepten – unter strenger Indikationsstellung – wird zwischenzeitlich auch im tagesstationären Setup durchgeführt [25].

Die bisherige Fast-Track-Literatur konzentrierte sich auf Aufenthaltsdauer („length of stay“ [LOS]) und stationäre Wiederaufnahmeraten [6–8] – Funktion, Schmerz und Mobilität wurden jedoch kaum untersucht.

## Material und Methoden

**Figure 1:**
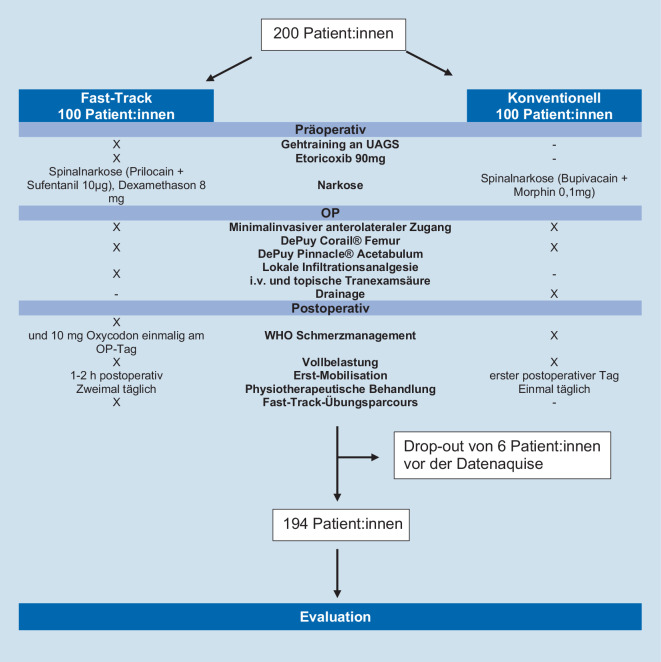

Wir führten eine wissenschaftliche Studie in einem kontrollierten Studiendesign durch [11]. Wir stellten die Hypothese auf, dass Fast-Track-Patienten nach HTEP bei vergleichbarem Schmerzniveau schneller und besser mobil sind. Es handelt sich um Teilergebnisse einer einfach verblindeten prospektiven, randomisierten, kontrollierte klinische Studie (RCT) zur Beurteilung der Mobilisierung und Mobilität, die den weit verbreiteten Timed-Up-and-Go-Test (TUG in Sekunden) [17] nach Fast-Track-HTEP im Vergleich zu einem konventionellen Behandlungspfad evaluierte. Darüber hinaus wurden Schmerzniveau (NRS) und die Mobilität (Gehstrecke in Meter) nach der Operation bewertet. Es wurden 200 Patienten eingeschlossen, die sich zwischen Mitte 2019 und Ende 2021 in unserer Einrichtung einer primären HTEP unterzogen. Alle eingeschlossenen Patienten wurden von einer unabhängigen Person randomisiert. Es wurde eine Blockrandomisierung mit vier Blöcken zu je 50 Patienten durchgeführt, gruppiert nach den beiden genannten Pfaden. Nur der Chirurg wusste, welcher Gruppe der Patient zugeordnet wurde (einfach verblindet), dies hatte regulatorische Gründe (Arzneimittelvorschriften in Deutschland für den Einsatz von lokaler Infiltrationsanalgesie, Tranexamsäure-Applikation). Darüber hinaus wurden die Fast-Track-Gruppe und die konventionelle Gruppe auf unterschiedlichen Stationen behandelt, um Kontakt und Austausch zu vermeiden. Einschlusskriterien waren: elektive primäre HTEP aufgrund primärer oder sekundärer Arthrose. Ausschlusskriterien waren: Alter < 18 Jahre oder > 90 Jahre, schwere Dysplasie der Hüfte, Verwendung anderer als der unten genannten Endoprothesenkomponenten, schwere Adipositas (BMI > 40 kg/m^2^), Tumorerkrankungen, Zustand nach tiefer Venenthrombose, therapeutische Antikoagulation, Immobilität bei präoperativer Gehstrecke < 100 m mit Unterarmgehstütze (UAGS) oder Rollator, eine frühere Fraktur im Operationsgebiet, offene Voroperation, gleichzeitige Teilnahme an einer anderen Studie oder Ablehnung durch den Probanden. Die Studie wurde von der lokalen Ethikkommission genehmigt (# 19-1308-101). 6 Patienten zogen ihr Einverständnis nach der Randomisierung, aber vor der Datenerhebung mit der Begründung zurück, dass die Datenauswertung zu umfangreich sei. Kein Patient verstarb innerhalb des Studienzeitraums. 194 Patienten wurden abschließend untersucht. Das Flussdiagramm (Abb. 1) zeigt die Details hinsichtlich der Unterschiede der Studienarme auf.

### Interventionen

Alle Fast-Track-Patienten erhielten präoperativ ein interdisziplinäres Seminar und ein physiotherapeutisch angeleitetes Gangtraining an UAGS. Darüber hinaus wurde Etoricoxib 90 mg einmal eine Stunde vor der Operation als präventive Analgesie angewendet. Die Fast-Track-Gruppe wurde in Spinalanästhesie operiert (Prilocain 1 % hyperbar 4 ml = 80 mg und Sufentanil 10 μg als Standard), mit intravenöser Verabreichung von Dexamethason (8 mg) [46]. Im Gegensatz dazu erhielt die Kontrollgruppe eine langwirksame Spinalanästhesie (Bupivacain 0,5 % = 4 ml und 0,1 mg Morphin).

In beiden Gruppen wurden in allen Fällen ein minimal-invasiver anterolateraler Zugang angewandt und eine zementfreie HTEP (DePuy Corail® Femurschaft, DePuy Pinnacle® Azetabulumkomponente, DePuy Orthopaedics, Warschau, IN, USA) verwendet. Im Gegensatz zur konventionellen Gruppe erhielt die Fast-Track-Gruppe sowohl periartikulär als auch subkutan eine Infiltrationsanalgesie (2 × 50 ml 0,2 % Ropivacain, für tiefe Infiltration mit 0,5 mg Adrenalin, LIA). Darüber hinaus wurde 1 g Tranexamsäure nach der Präparation topisch verabreicht und weitere 2 g intraartikulär gegen Ende des Eingriffs durch die verschlossene Faszie appliziert. Ein weiterer Unterschied in der Fast-Track-Gruppe war der Verzicht auf Drainagen, das Auftragen von Wundkleber nach dem Wundverschluss und die Verwendung einer transparenten Wundauflage. In beiden Gruppen war die Vollbelastung ohne Restriktionen erlaubt. In der Fast-Track-Gruppe begann die Mobilisierung, sobald die periphere sensomotorische Funktion wiedererlangt war, üblicherweise 1–2 h nach der Operation.

Nach kreislaufanregenden und Thromboseprophylaxe-Übungen wurden unter physiotherapeutischer Aufsicht erste Gehübungen an UAGS durchgeführt. Das Ziel für den Operationstag war eine Gehstrecke von mindestens 50 Metern. Im Gegensatz dazu begann die Mobilisierung nach konventioneller HTEP ab dem ersten postoperativen Tag, nachdem die anhaltenden sensomotorischen Funktionseinschränkungen nachgelassen hatten. Drainagen wurden am zweiten postoperativen Tag entfernt.

Anschließend wurde ein standardisiertes Physiotherapiekonzept zweimal täglich in der Fast-Track-Gruppe und einmal täglich in der konventionellen Gruppe durchgeführt. Die Physiotherapie umfasste Mobilisierung, Muskelstärkung, Thrombose- und Lungenentzündungsprävention. Ab dem ersten postoperativen Tag wurde für die Patienten der Fast-Track-Gruppe ein Fast-Track-Parcours genutzt, um die Intensität der Bewegung und des Trainings zu steigern; die Patienten erhielten zusätzlich zweimal täglich Physiotherapie. Der Fast-Track-Übungsparcours besteht aus Gangtraining, verschiedenen Übungen zur Muskelstärkung und Anleitungen zur Verbesserung der Koordination. Darüber hinaus kam auf der Fast-Track-Station regelmäßig eine Spiegelwand mit Stützstange zum Einsatz. Dabei konnten die Patienten die erlernten Übungen mehrmals täglich selbstständig und unter Selbstkontrolle wiederholen, um ihr Gangbild zu reflektieren und mögliche Fehler selbst zu korrigieren. Nach der Operation wurden den Probanden in der Fast-Track-Gruppe einmal am Operationstag 10 mg Oxycodon verabreicht. In unserer Abteilung wurde ein standardisiertes Schmerzmanagementkonzept gemäß den Empfehlungen der Weltgesundheitsorganisation (WHO) [18] erstellt: Die orale Schmerzmedikation besteht aus Ibuprofen (600 mg) 3‑mal täglich und Metamizol (500 mg) 4‑mal täglich. Abhängig von den NRS-Werten können Patienten bei Bedarf Tramadol 100 mg (40 Tropfen) oder Oxycodon 10 mg als optionale zusätzliche schmerzstillende „rescue medication“ erhalten. Auf der Aufwachstation wurden optional je nach Bedarf 3 mg Piritramid verabreicht, abhängig von der NRS. Um das verblindete Studiendesign und die standardisierte Vergleichbarkeit zu gewährleisten, wurden alle Patienten nach der Operation für eine Woche stationär und auf getrennten Stationen behandelt.

Der weit verbreitete und standardisierte TUG [19] wurde präoperativ und postoperativ täglich durch einen unabhängigen Untersucher durchgeführt. Alle Studienpatienten führten den Test einmal durch. Die Zeitmessung umfasste die Zeit vom Aufstehen vom Stuhl, dem Gehen von drei Metern, dem Umdrehen, dem Zurückgehen zum Stuhl und dem Hinsetzen. Darüber hinaus wurde die Gehstrecke in Metern gemessen. Um Schmerz zu erfassen, wurde ein subjektiver Fragebogen verwendet. Es wurde ein Mittelwert aus Ruhe‑, Mobilisierungs- und Nachtschmerzen gebildet, präoperativ und täglich postoperativ. Die Schmerzen wurden anhand der NRS von 0 (= überhaupt keine Schmerzen) bis 10 (= schlimmste jemals mögliche Schmerzen) beurteilt.

### Statistik

Im Vorfeld wurde eine Power-Berechnung durchgeführt (basierend auf den retrospektiven Ergebnissen der ersten 100 Fast-Track-Patienten der Abteilung bezüglich der designierten primären Ergebnisvariablen TUG, *p* < 0,05): *n* = 100 Patienten pro Studienarm, um eine Power von 0,8 mit einer erwarteten Dropout-Quote von 5 % zu erreichen.

Vergleiche zwischen beiden Behandlungsgruppen wurden entweder mithilfe des Student-t-Tests oder des Wilcoxon-Mann-Whitney-Tests für kontinuierliche Daten oder des Chi-Quadrat-Unabhängigkeitstests für kategoriale Daten durchgeführt. Ein p‑Wert < 0,05 wurde für alle Tests als statistisch signifikant angesehen. Alle Analysen wurden mit Statistical Package for the Social Sciences (IBM SPSS Statistics for Windows, Version 27.0, IBM Corp., Armonk, NY, USA) und R (v4.2.1; R Core Team 2021) durchgeführt.

## Ergebnisse

Insgesamt wurden 194 Patienten untersucht, die sich einer HTEP unterzogen (98 Fast-Track/96 konventionell). Das Durchschnittsalter betrug 64,31 Jahre (± 9,87) für Fast-Track und 65,55 Jahre (± 8,45) für die konventionelle Gruppe (nicht signifikant, n. s.). In beiden Studiengruppen wurden innerhalb der ersten Woche postoperativ keine Komplikationen wie thromboembolische Ereignisse, Frakturen oder Revisionen verzeichnet.

**Figure 2:**
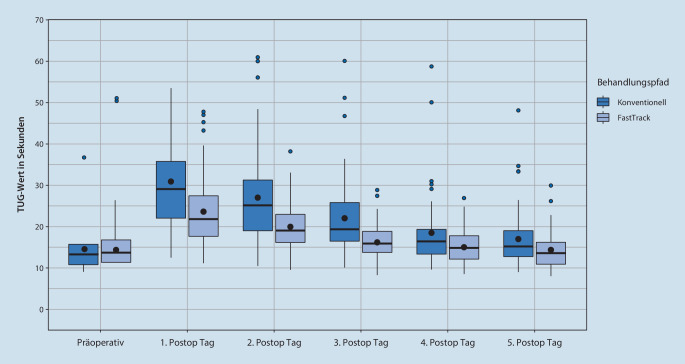

Vor der Operation konnten keine signifikanten Unterschiede zwischen beiden Gruppen festgestellt werden. Die Fast-Track-Gruppe zeigte nach der Operation bis zum sechsten postoperativen Tag im Vergleich zur konventionellen Gruppe signifikant (*p* < 0,05) bessere TUG- und Gehstreckenergebnisse (Abb. 2 und 3).

**Figure 3:**
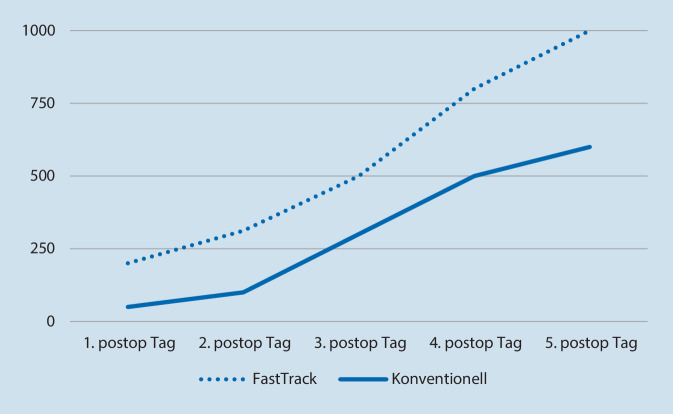

**Figure 4:**
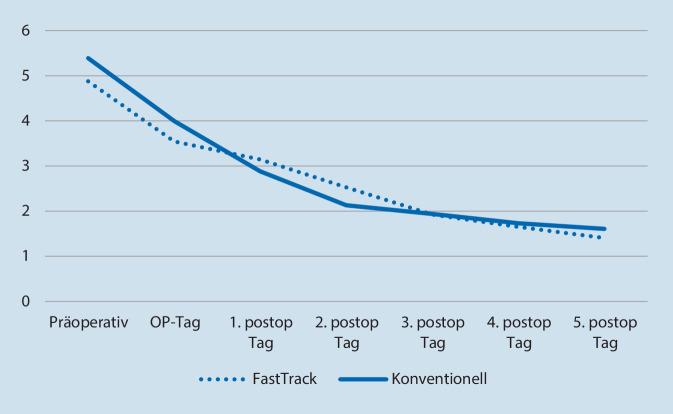

Hinsichtlich prä- und postoperativer Schmerzen (NRS 0–10, Abb. 4) wurde kein signifikanter Unterschied zwischen Fast-Track und dem konventionellen Weg beobachtet (n. s.). Alle Schmerzwerte waren im postoperativen Verlauf rückläufig und unterschritten bereits am Operationstag das präoperative Niveau.

## Diskussion

Die Daten dieser einfach verblindeten RCT-Studie zu einem Fast-Track-Vorgehen im Vergleich zu einem konventionellen Behandlungspfad nach HTEP über einen Zeitraum von einer Woche zeigen eine signifikant bessere Mobilisation (TUG, primäre Ergebnisvariable, *p* < 0,05) und eine weitere Gehstrecke bei vergleichbaren Schmerzen, einem sehr guten kurzfristigen funktionellen Ergebnis entsprechend.

Der TUG-Test wurde in vielen relevanten Studien zur Hüftendoprothetik eingesetzt [21, 22], in der Literatur wurde jedoch keine vergleichbare Fast-Track-Studie hinsichtlich TUG, Schmerz und Gehstrecke gefunden. Nach Fast-Track-HTEP konnten wir in der ersten postoperativen Woche eine deutlich bessere Mobilität (TUG-Test, *p* < 0,05) erreichen. Bei Fast-Track-Behandlungspfaden gilt die frühe Mobilisierung als ein wesentlicher Baustein [23]. Wainwright et al. empfahlen in einer Konsenserklärung die schnellstmögliche Mobilisierung von Fast-Track-Patienten nach der Operation [24], wie auch die Dänen Hustet und Kehlet [22]. Eine frühzeitige Mobilisierung muss immer mit einer guten Balance einhergehen, um das Sturzrisiko der Patienten zu verringern [26]. Nach einer HTEP stürzten in einer Analyse 23,2 % der Menschen in den ersten 12 Monaten nach der Operation [25], dies zeigt die Wichtigkeit einer raschen und sicheren Erholung nach der Operation im Rahmen von Fast-Track-Konzepten. Ein wesentliches Instrument zum Screening von Mobilität und Gleichgewichtsdefiziten ist der TUG-Test [26]. Wie durch eine univariate Regressionsanalyse analysiert wurde, war der TUG-Test auch signifikant mit LOS assoziiert [27]. Wir konnten nachweisen, dass eine frühe Mobilisierung im Rahmen eines Fast-Track-Behandlungspfades zu einem signifikant (*p* < 0,05) besseren TUG-Ergebnis im postoperativen Verlauf führt. Wir stellten in der Fast-Track-Gruppe eine signifikant (*p* < 0,05) erhöhte Gehstrecke bei vergleichbaren Schmerzen (*p* < 0,05) fest. Bereits am zweiten postoperativen Tag konnten Fast-Track-Patienten 252 m gehen, während Patienten in der konventionellen Gruppe nur 120 m zurücklegen konnten (*p* < 0,05), während das Schmerzniveau bereits am Operationstag die präoperativen Werte unterschritt. Frühere Studien haben bereits die Bedeutung „beschleunigter“ physiotherapeutischer Behandlungsprotokolle gezeigt. Hierdurch waren bessere Ergebnisse im Gang und in der Muskelkraft möglich [29]. Eine intensivierte aktive Behandlung und zusätzliche Mobilisierung, die bei Fast-Track bereits am Operationstag nach HTEP begann, erzielten bessere Ergebnisse. Insgesamt handelt es sich bei Fast-Track nicht nur um eine verstärkte Physiotherapie – Fast-Track ist ein komplexes multidisziplinäres Setup, wobei einzelne „Behandlungsbausteine“ nicht zum Behandlungserfolg führen werden.

Den Patienten ist in der Regel unmittelbar nach der HTEP die volle Belastung gestattet, dies kann jedoch die postoperativen Schmerzen verstärken und folglich die Genesung und Rehabilitation beeinträchtigen [32]. Daher scheint die multimodale Schmerzbehandlung auch ein entscheidender Faktor für die operative Versorgung von Patienten mit HTEP zu sein. Eine sorgfältige Beurteilung und Behandlung der Schmerzen sind bei Fast-Track von entscheidender Bedeutung, um postoperative Komplikationen und eine Einschränkung in der Mobilität zu vermeiden [33]. Zu einer optimalen Schmerztherapie gehört auch die intraoperative Anästhesie. Eine im Jahr 2018 durchgeführte Metaanalyse zeigte, dass die besten Ergebnisse nach einer HTEP in Bezug auf die Schmerzbehandlung mit einer Spinalanästhesie in den ersten 24 h erzielt wurden [34]. Die interdisziplinäre Prozessoptimierung aller Behandlungspfade führte nicht nur zu einer besseren Mobilisierung, sondern auch zu geringeren Schmerzscores. Obwohl Fast-Track-Patienten in unserer Studie mehr Physiotherapie und ein intensiveres Mobilisierungstraining erhielten, haben wir während des gesamten Beobachtungszeitraums vergleichbare Schmerzniveaus wie bei Patienten mit konventionellem Vorgehen beobachtet.

Okamoto et al. bestätigten, dass die Gesamtheit der Bausteine des Fast-Track-Programms wichtig sind und in der Kombination zu einer Verringerung der Komplikationen führten [35]. Da es auch in unserem Beobachtungszeitraum zu keinen Komplikationen kam, kann unser Fast-Track-Programm als ein für den Patient sicheres und risikofreies Konzept gewertet werden.

## Limitationen

Die vorliegende Studie wurde aufgrund der Vorschriften in Deutschland in einem einfach verblindeten Studiendesign durchgeführt (Medikamente – LIA und Tranexamsäure wurden intraoperativ nur in der Fast-Track-Gruppe verwendet), daher musste der Chirurg wissen, welcher Studiengruppe die Patienten zugeordnet wurden. Dennoch wurden die Daten von einem unabhängigen Team aus Arzthelferinnen und Physiotherapeuten ausgewertet, sodass Verzerrungen auf ein absolutes Minimum reduziert werden sollten. Zweitens wurden immobilisierte Patienten in dieser Studie ausgeschlossen, sodass unklar bleibt, wie sich Fast-Track-HTEP auf den Erfolg eines älteren immobilen Patientenkollektivs mit Komorbiditäten auswirkt – aktuell Gegenstand weiterer wissenschaftlicher Untersuchungen im Rahmen eines GBA-Projektes. Um standardisierte und kontrollierte Studienbedingungen zu gewährleisten, wurde die Nachbeobachtungszeit in unserer Studie auf eine Woche begrenzt, sodass mittel- oder langfristige Ergebnisse derzeit nicht verfügbar sind. Dennoch könnte auch die kurze Nachbeobachtungszeit als Stärke der Studie bezeichnet werden, da der klare und standardisierte Aufbau mit zwei Armen und gleichem LOS unter Berücksichtigung von HTEP in den Gesundheitssystemen anderer Länder nicht zu finden ist. Mit dieser aufwendigen prospektiven randomisierten Studie können qualitative Rückschlüsse auf das postoperative Outcome gezogen werden. Die meisten qualitativ hochwertigen Studien zu diesem Thema untersuchten die Verweildauer (LOS), aber bisher lagen keine Daten zu Mobilisierung, Funktion und Schmerz vor – was uns zur Durchführung der Studie veranlasst hat. Zukünftig sollten prospektive multizentrische Langzeitstudien in einem randomisierten, kontrollierten Studiendesign durchgeführt werden, um das in dieser Studie erworbene Wissen zu erweitern und Schmerzen, funktionelles Ergebnis und Verbesserungen der Lebensqualität nach Fast-Track-HTEP zu messen und zu bewerten.

## Fazit

Diese prospektive, einfach verblindete, randomisierte, kontrollierte klinische Studie belegte, dass Fast-Track-Konzepte hinsichtlich des klinischen Ergebnisses äußerst effektiv sind und erfolgreich in der täglichen klinischen Praxis eingesetzt werden können. Fast-Track-Patienten nach zementloser HTEP zeigten signifikant bessere TUG-Werte sowie besser tägliche Mobilität, während das Schmerzniveau zum konventionellen Vorgehen vergleichbar war. Während einer Nachbeobachtungszeit von einer Woche sanken die Schmerzwerte kontinuierlich. Aufgrund der guten Ergebnisse werden in unserer Abteilung inzwischen alle Patienten nach dem Fast-Track-Protokoll behandelt, auch fragile und multimorbide Patienten.

## Fazit für die Praxis

- Fast-Track-Konzepte bei Hüft- und Kniegelenkersatzoperationen finden zunehmend auch in Deutschland Anwendung.
- Mit Fast-Track kann eine rasche Rekonvaleszenz für die Patienten erreicht werden, die frühpostoperative Mobilisation steht im Fokus des Behandlungskonzeptes.
- Möglich wird dies nur durch optimierte Behandlungspfade in enger interdisziplinärer Zusammenarbeit sowie der Implementierung spezifischer Fast-Track-Bausteine, welche nur im „Gesamtpaket“ funktionieren.
- Wesentliche perioperative Bausteine sind:
  - präoperative Seminare und physiotherapeutisches Training
  - präemptive Analgesie, schonende kurzwirksame Narkosetechniken, gewebeschonende minimal-invasive Operationstechniken
  - postoperative schnellstmögliche Mobilisation
- Für die primäre Fast-Track-Hüfttotalendoprothetik können wir in einem randomisierten Setup eine signifikante Überlegenheit hinsichtlich postoperativer Mobilisation und erreichbarer Gehstrecke bei vergleichbarem Schmerzniveau und Komplikationsrisiko zeigen.

## Abkürzungen

ADL: Activities of Daily Living
BMI: Body-Mass-Index
HTEP: Hüfttotalendoprothese
LIA: Lokale Infiltrationsanalgesie
LOS: „Length of stay“
NRS: Numerische Rating-Skala
PONV: „Postoperative nausea and vomiting“
RCT: „Randomized controlled trial“
TUG: Timed-Up-and-Go-Test
UAGS: Unterarmgehstützen
